# Poly Lactic-*co*-Glycolic Acid-Coated Toluidine Blue Nanoparticles for the Antibacterial Therapy of Wounds

**DOI:** 10.3390/nano11123394

**Published:** 2021-12-14

**Authors:** Xiaomu Xu, Bo Liu, Haiyan Wu, Yichi Zhang, Xinyuan Tian, Jijing Tian, Tianlong Liu

**Affiliations:** Laboratory of Veterinary Pathology and Nanopathology, College of Veterinary Medicine, China Agricultural University, No. 2 West Road Yuanmingyuan, Beijing 100193, China; xuxiaomu92@126.com (X.X.); liubo19900409@163.com (B.L.); wuhaiyan_helen@sina.com (H.W.); zhangyichi142857@163.com (Y.Z.); tianxy@cau.edu.cn (X.T.)

**Keywords:** photodynamic therapy, toluidine blue nanoparticles, wound healing, antibacterial

## Abstract

Bacterial infections in wounded skin are associated with high mortality. The emergence of drug-resistant bacteria in wounded skin has been a challenge. Toluidine blue (TB) is a safe and inexpensive photosensitizer that can be activated and used in near-infrared photodynamic therapy to effectively kill methicillin-resistant *Staphylococcus aureus* (MRSA). However, its aggregation-induced quenching effect largely affects its clinical applications. In this study, TB nanoparticles (NPs) were synthesized using an ultrasound-assisted coating method. Their physicochemical and biological properties were studied and evaluated by scanning electron microscopy and Fourier-transform infrared spectroscopy. The TBNPs had a broad-spectrum antibacterial activity against Gram-positive bacteria (MRSA) and Gram-negative bacteria (*E. coli*). In addition, MTT, hemolysis, and acute toxicity tests confirmed that TBNPs had good biocompatibility. The TBNPs exhibited a high photodynamic performance under laser irradiation and efficiently killed *E. coli* and MRSA through generated reactive oxygen species, which destroyed the cell wall structure. The potential application of TBNPs in vivo was studied using an MRSA-infected wound model. The TBNPs could promote wound healing within 7 days, mainly by reducing the inflammation and promoting collagen deposition and granulation tissue formation. In conclusion, the TBNPs offer a promising strategy for clinical applications against multiple-drug resistance.

## 1. Introduction

The skin is the largest organ of the body. Its primary function is to protect against pathogenic microbial infections, chemical agents, and mechanical damage [1,2]. When skin is damaged by external agents or microbes, its function as a barrier against organisms is affected, in addition to its temperature regulation and immune regulation abilities [3]. Once wounded, bleeding, inflammation, and bacterial infection may occur [4]. It is, therefore, challenging to cure wounds; in particular, the emergence of drug-resistant microorganisms, which further complicates the situation, makes it more arduous [5]. Drug-resistant microorganisms, such as methicillin-resistant *Staphylococcus aureus* (MRSA), have become a major health care concern worldwide [6,7,8]. Approximately half of all skin infections arise due to MRSA. Therefore, a multi-functional antimicrobial agent is needed to overcome this problem.

Recently, photodynamic therapy (PDT), a novel antimicrobial treatment, has attracted increasing attention in clinical applications and animal disease models [9,10,11] because of its unique antibacterial mechanism, which is less likely to cause drug resistance in bacteria [12,13]. A hundred years ago, PDT was used to treat various skin diseases, such as skin cancer and acne [14,15,16]. PDT has become an alternative tool to replace antibacterial drugs. Light, oxygen, and a photosensitizer are the important factors in PDT. It is generally believed that the bactericidal mechanism of PDT involves the production of reactive oxygen intermediates (such as singlet oxygen and hydrogen peroxide) under light exposure, eventually leading to cell death by apoptosis or necrosis [17,18].

**Scheme 1:**
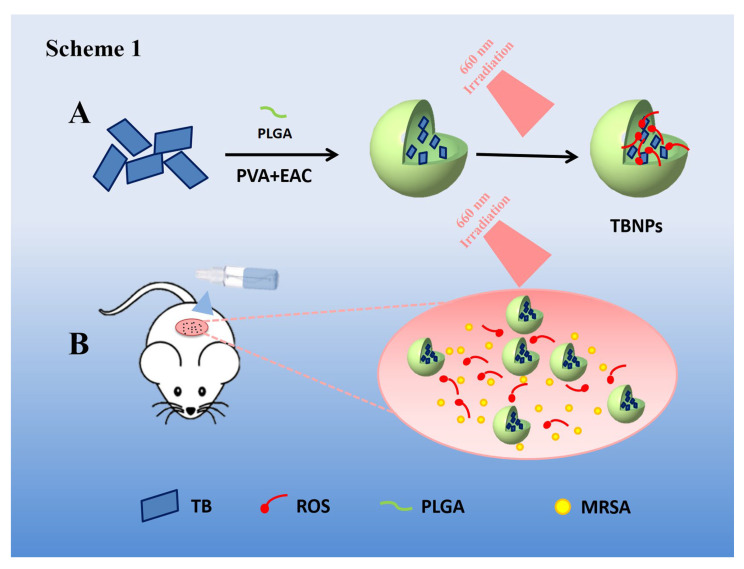
Experiment diagrams. (**A**) Schematic of the synthetic route of TBNPs. (**B**) Schematic of TBNPs producing an increased number of reactive oxygen species (ROS) under 660 nm laser irradiation in a wound.

In this study, a nanospray was synthesized, and PDT was used to treat wounds infected by bacteria. Toluidine blue (TB) is a conventional photosensitizer. TB-mediated PDT has good antibacterial and healing properties against bacterial keratitis and skin infections [19]. However, the clinical application of TB is limited mainly because of the aggregation-induced quenching (ACQ) effect. In addition, it is challenging to maintain the target site for a long time [20,21]. Therefore, TB was modified with poly lactic-*co*-glycolic acid (PLGA) to improve its antibacterial effect. PLGA is a macromolecular organic compound with good biocompatibility and biodegradability [22]. PLGA is widely used as a biomedical material for various applications, including drug carriers, medical/surgical materials, and tissue engineering [23,24,25]. It has been approved by the Food and Drug Administration (FDA) as a clinical wound dressing. PLGA promotes angiogenesis and accelerates wound healing [26,27]. Multi-functional TB nanoparticles (NPs) were prepared for the treatment of MRSA infection (Scheme 1). PLGA-coated TBNPs were prepared to treat *E. coli* and MRSA infections. The conventional plate count method and minimum inhibitory concentration (MIC) test were used to evaluate the antibacterial effect of TBNPs in vitro. In addition, the therapeutic effects of TBNPs on skin infections caused by MRSA were evaluated in mice.

## 2. Materials and Methods

**Preparation of TBNPs.** A TB solution (10 mg/mL) with 3% NaCl was used as an internal aqueous phase. PVA solutions of concentrations 1% and 0.5% were configured as external aqueous phases. PLGA (200 mg) was dissolved in 5.5 mL of ethyl acetate, and 0.5 mL of the internal aqueous phase was added into oil phase. Colostrum was formed by ultrasound in the ice bath and was poured into 10 mL of the 1% PVA external aqueous phase. The premixed milk was prepared in an ultrasonic ice bath for 10 min (operation for 3 s, pause for 3 s). The premixed milk was poured into 10 mL of the 0.5% PVA solution and stirred at 300–400 rpm at room temperature for 3–4 h, which yielded PLGA NPs. The solution was then centrifuged at 4 °C at 10,000 rpm for 10 min. Distilled water was added for ultrasonic dispersion.

**Characterization of TBNPs**. The hydrodynamic size and potential of the NPs were measured using a Zetasizer nanoparticle size potentiometer (Zetasizer Nano ZS90; Malvern, UK). The morphology of the TBNPs was observed using scanning electron microscopy (SEM) (FEI Inspect F50, Hillsboro, OR, USA). The chemical structure of the TBNPs was analyzed at the molecular level using Fourier-transform infrared (FTIR) spectroscopy.

**Minimum Inhibitory Concentration Test.** MRSA (ATCC 25923) and *E. coli* (ATCC 25922) were used for MIC detection. A 5 × 10^5^ colony-forming unit (CFU)/mL bacterial suspension of 100 μL was added to each well. TBNPs with different concentrations were added to each well in volumes of 100 μL. The light groups were illuminated at 660 nm for 5 min and incubated at 37 °C for 18 h. Resazurin of 0.0675% concentration was then prepared, and 10 μL of it was added to each well and incubated at 37 °C for 4 h. Color changes were observed, and any well indicating color change from purple to pink or colorless was recorded as positive.

**MTT Test.** The cytotoxicity of TB and TBNPs was evaluated by an MTT viability assay. The light groups were illuminated at 660 nm for 5 min. 4T1 cells were maintained in a high-glucose Dulbecco’s modified Eagle’s medium. After coincubating the 4T1 cells (5 × 10^4^/well) with TBNPs for 24 h at a series dosage, MTT solution was added to each well. After 4 h of incubation at 37 °C, colorimetric measurements were performed at 495 nm using a scanning multi-well spectrometer.

**In Vivo Biocompatibility Assay.** Mice were divided into different groups (PBS, PBS + light, TB, TB + light, TBNPs, and TBNPs + light; 5 mice in each group) and treated via the tail vein. The hearts, livers, spleens, lungs, and kidneys of the mice were harvested for a histological analysis to evaluate the biocompatibility of the TBNPs.

**In Vitro Antibacterial Evaluation.** MRSA and *E. coli* were used to investigate the antibacterial effects of the TBNPs for six groups (PBS, PBS + light, TB, TB + light, TBNPs, and TBNPs + light (50 mg/kg)). Bacterial suspensions at a concentration of 1.5 × 106 CFU/mL were added to a 96-well plate and then treated under the above conditions for 2 h at 37 °C. Groups treated with the 660 nm laser were separately irradiated with a 660 nm laser for 5 min (1 W/cm^2^). The mixed solution of each treatment group was coated on a solid agar plate and cultured at 37 °C for 24 h. Morphological changes in bacteria (MRSA and *E. coli*) treated with TBNPs were observed by SEM.

**Detection of Single Oxygen.** TB and TBNPs were dispersed in a water solution for single-oxygen tests. The trapping agent used in this test was ABDA, which was pipetted into the solution and mixed homogeneously for preparation. Capillaries were then introduced to load the samples. The capillaries were then placed into an electron paramagnetic resonance (EPR) system (JEOL, JES-FA200, Tokyo, Japan) to collect information on the radicals. Electron spin resonance (ESR) spectra were recorded using a Bruker E500 spectrometer (Karlsruhe, Germany). A mixture of the TBNPs and singlet oxygen (^1^O_2_) trapper (TEMP) in an aqueous solution was transferred to a standard quartz capillary and irradiated by the 660 nm laser (1 W/cm^2^) for different time intervals (0, 4, 8, 12, and 16 s) to monitor ^1^O_2_.

**Animal Anti-Infection Model Assay.** Female BALB/c mice were selected to establish a skin infection animal model to evaluate the antibacterial potential of TBNPs. The skin on the back was punctured with a sterile syringe needle, inoculated with 5 × 107 CFU/mL MRSA (100 μL), and infected for one day. The mice successfully infected with MRSA were randomly divided into six groups (three in the light group and three in the nonlight group), with five mice in each group. PBS, TB, and TBNPs were sprayed into the infected area. For the light group, the treatment was carried out at 5 min after spraying. After 24 h, 3 days, and 7 days, clinical examination, bacterial culturing, and biochemical identification were performed. Colonies were enumerated and reported as recovered CFU/mL of washing fluid. The skin tissue from each mouse was fixed in a 10% buffered formalin phosphate at room temperature followed by paraffin embedding. Histological slide preparation and hematoxylin–eosin (H–E) staining were performed. The collected H–E-stained mouse histology sections were visualized using an optical microscope (Olympus X71, Tokyo, Japan). All identities and analyses of the pathology slides were blinded to the pathologist.

**Statistical analysis.** The data are presented as mean ± standard deviation. A statistically significant difference was defined by *p* < 0.05.

## 3. Results

### 3.1. Preparation and Characterization of TBNPs

**Figure 1:**
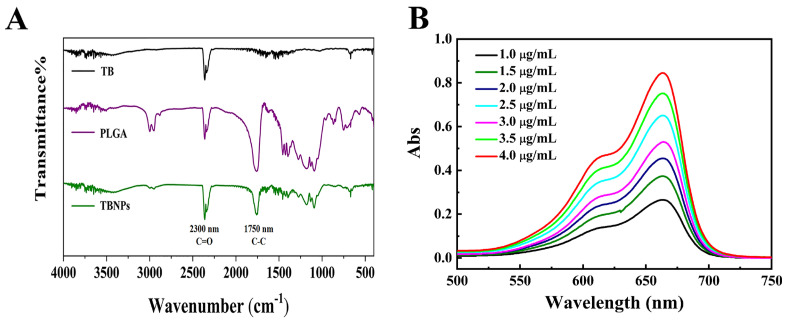
(**A**) FTIR spectra of TB, PLGA, and TBNPs. (**B**) Ultraviolet–visible absorption spectra of TBNPs.

The performances of the TBNPs were evaluated by SEM and FTIR spectroscopy. Compared to TB, the FTIR spectrum of the TBNPs showed new absorption bands at 2300 and 1750 nm^−1^. The absorption band at 2300 cm^−1^ was attributed to the C=O stretching vibration, while the absorption band at 1750 cm^−1^ was attributed to the bending vibration of the C–C skeleton of the benzene ring of TB (Figure 1A). The average size of the TBNPs measured by dynamic light scattering was 201.7 nm with a polydispersity index of 0.074 (Figures S1 and S2). The zeta potential of the TBNPs was −8.14 mV (Figure S3). The morphology of the TBNPs was observed by SEM. The TBNPs had a uniform appearance with size in the range of 100–200 nm (Figure S4). The polylactic acid successfully coated the TB. Ultraviolet–visible absorption spectroscopy revealed that the TBNPs exhibited a wide absorption range (ultraviolet to near-infrared) at different concentrations. As shown in Figure 1B, the maximum absorption peak of the TBNPs is 663.5 nm, which is the characteristic wavelength of TB. As shown in Figure S5, a linear regression equation of *y* = 0.1975*x* − 0.00669 (R^2^ = 0.99961) is obtained considering the TB concentration C (μg/mL) as abscissa and the absorbance of the TB solution at 660 nm as the ordinate. The TBNP encapsulation rate and drug load of the NPs were 87.03% and 2.24%, respectively.

### 3.2. Hemolysis Test

A hemolysis test was used to evaluate the interaction between the nanocomposite materials and blood. When the concentration of the TBNP solution reached 400 µg/mL, no significant hemolysis occurred (Figure S6), which indicates a good biocompatibility. As shown in Figure S7, the concentration of the TBNP solution reaches 400 µg/mL, which corresponds to a rare hemolysis. The red blood cell morphology was normal, without hemolysis (Figure S8).

### 3.3. Phototoxic Cell Assay

**Figure 2:**
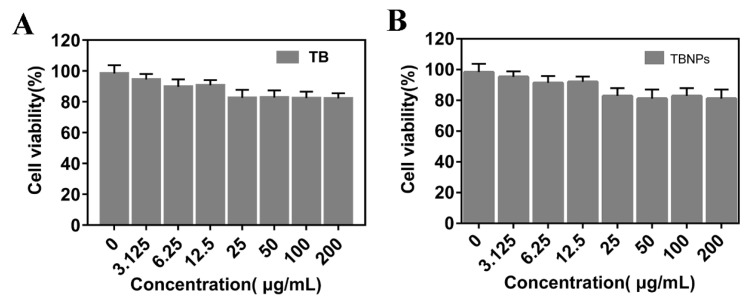
Cell viability experiment. Activities of 4T1 cells incubated with (**A**) TB and (**B**) TBNPs at different concentrations after 660 nm laser radiation for 5 min.

Although TB had a very low toxicity after 5 min of 660 nm laser radiation, the physiological behavior of the TBNPs may be significantly different when TB is modified with PLGA. Therefore, the toxicology of the TBNPs requires further evaluation. Different concentrations of TB and TBNPs (0, 3.125, 6.25, 12.5, 25, 50, 100, and 200 μg/mL) were cultured in 4T1 cells after 5 min of 660 nm laser irradiation for 24 h. The cell viability was evaluated by an MTT assay. The cell viability of TB at concentrations of 3.125, 6.25, 12.5, 25, 50, 100, and 200 μg/mL was 91.2%, 90.4%, 89.7%, 86.6%, 84.2%, 86.3%, and 82.6%, respectively (Figure 2A). The cell viability of the TBNPs at concentrations of 3.125, 6.25, 12.5, 25, 50, 100, and 200 μg/mL was 93.2%, 91.4%, 92.7%, 86.6%, 84.2%, 86.3%, and 84.6%, respectively (Figure 2B). The TB and TBNPs exhibited low phototoxicities in the case without irradiation (Figure S9).

The relative activity of 4T1 cells did not change significantly after incubation for 24 h with TB and TBNPs, even at 200 μg/mL, which indicates a low cytotoxicity of the tested nanomaterials. This demonstrates that the TBNPs have a low phototoxicity, which can be used in a series of cell experiments and are expected to be used further in animal experiments.

### 3.4. In Vivo Toxicity

**Figure 3:**
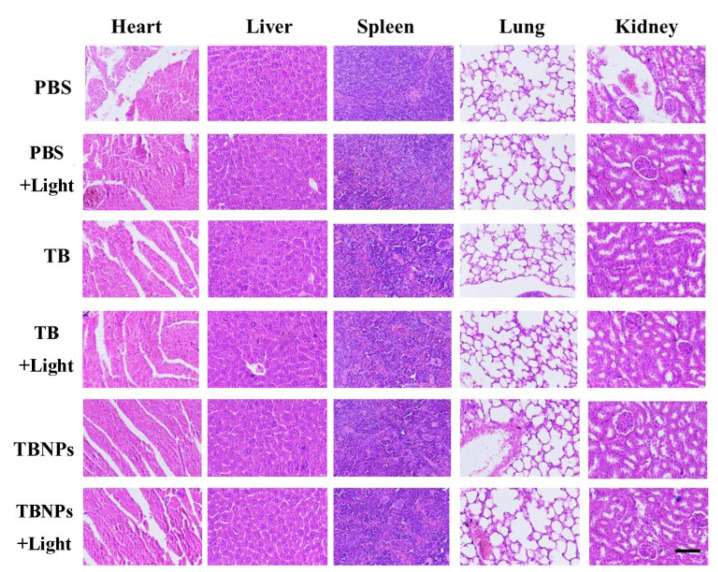
Photomicrographs of H–E-stained slides of the major organs from the mice 14 days after intravenous injection with PBS (100 μL), TB (200 μg/mL, 100 μL), and TBNPs (200 μg/mL, 100 μL). The scale bar corresponds to 500 μm.

Although the biocompatibility of TB has been reported, the toxicity evaluation of TBNPs in vivo has not been carried out. The biological safety of TBNPs was verified in mice. The histopathology with H–E staining indicated no noticeable damage or inflammation in the tissues (the heart, liver, spleen, lungs, and kidneys) from all groups (Figure 3). These results show that the TBNPs had a good biocompatibility in vivo.

### 3.5. In Vitro Antibacterial Treatment

**Figure 4:**
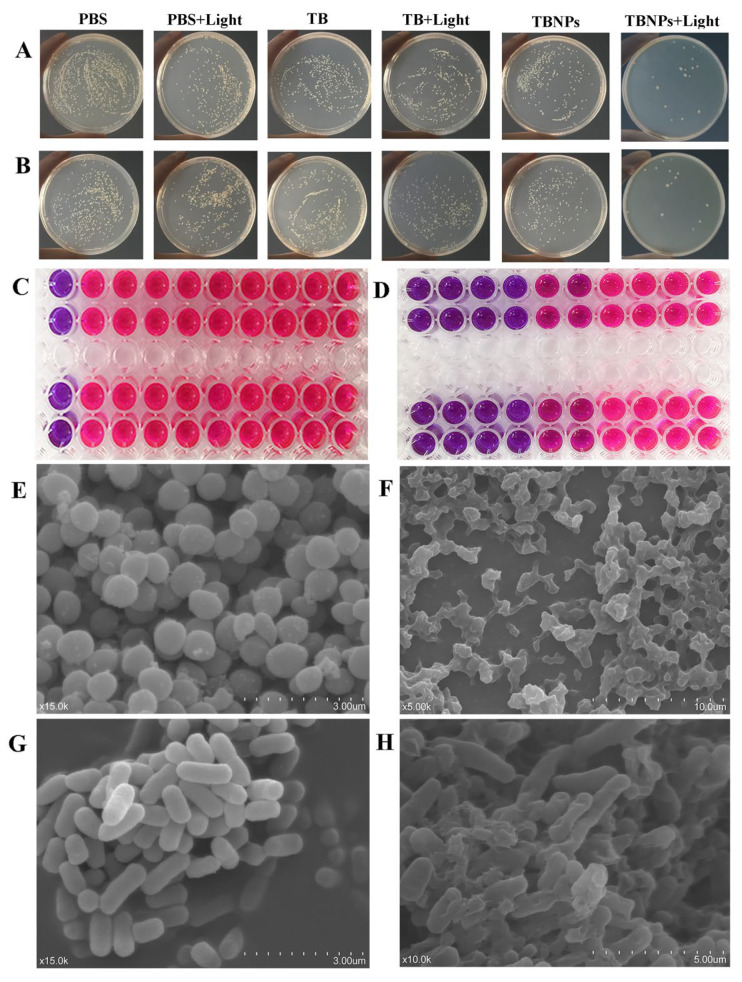
Photographs of bacterial colonies formed on LB agar plates of (**A**) *E. coli* and (**B**) MRSA (PBS, PBS + light, TB, TB + light, TBNPs, and TBNPs + light). (**C**) Image of the MIC of TB against *E. coli* and MRSA. (**D**) Image of the MIC of TBNPs with the 660 nm laser against *E. coli* and MRSA. SEM images of (**E**) MRSA in PBS, (**F**) MRSA treated with TBNPs under the laser, (**G**) *E. coli* in PBS, and (**H**) *E. coli* treated with TBNPs under the laser.

The antimicrobial activity of the TBNPs was evaluated using the MIC test. The MIC of the TBNPs for MRSA and *E. coli* was 100 μg/mL (Figure 4C). However, the MIC of TBNPs + light against *E. coli* (top row) and MRSA (next row) was 25 μg/mL (Figure 4D). The MIC results indicated that the TBNPs + light treatment had an obvious antibacterial effect.

To evaluate the antibacterial activity of the TBNPs by a single laser radiation, MRSA and *E. coli* were cultured with TBNPs, TB, and PBS before treatment with or without laser irradiation (660 nm, 1 W/cm^2^, 5 min). MRSA or *E. coli* colony counts were measured using the conventional plate count method after 24 h. As shown in Figure 4A,B, the TBNPs + light treatment produces the strongest antibacterial effect, with few colonies surviving in both strains (MRSA and *E. coli*) on the TBNPs + light-treated plates. In contrast, the colony counts in the PBS and TB treatment groups were relatively high regardless of the irradiation, which indicates that laser irradiation was not a significant factor limiting the bacterial growth. As shown in Figure S5, TB and TBNPs are largely limited, and 27.2% and 29.7% of MRSA were killed when the cells were treated with TBNPs and TB (200 µg/mL). However, TB + light exhibited a better antibacterial effect, with few colonies of MRSA surviving on light-treated plates (74.4%). In contrast, TBNPs + light exhibited an extremely high antibacterial efficiency (99%). For *E. coli*, the restrain rate after treatment with TBNPs plus laser was 98.9%, which was significantly higher than those in the other treatment groups (TB: 36.1%, TB + light: 80.6%, TBNPs: 34.6%, and PBS + light: 24.9%).

Morphological changes in *E. coli* and MRSA treated with TBNPs and 660 nm laser were observed by SEM. In the control group, intact cell morphology and smooth surfaces were observed in MRSA (Figure 4E) and *E. coli* (Figure 4G), respectively. Compared to the control group, in the TBNPs + light group, some *E. coli* cells were significantly wrinkled (Figure 4H); certain MRSAs adhered to each other, had different sizes, spillage of the contents occurred (Figure 4F); most of the bacteria were killed; and the normal morphology of bacteria disappeared. The significant improvement in the antibacterial activity of the TBNPs was attributed to the synergistic PDT/TBNPs. TBNPs bind to bacterial membranes. The PDT can destroy the outer membrane of bacteria by producing ROS, thereby enhancing the antibacterial properties.

### 3.6. Treatment of the MRSA-Infected Wound

**Figure 5:**
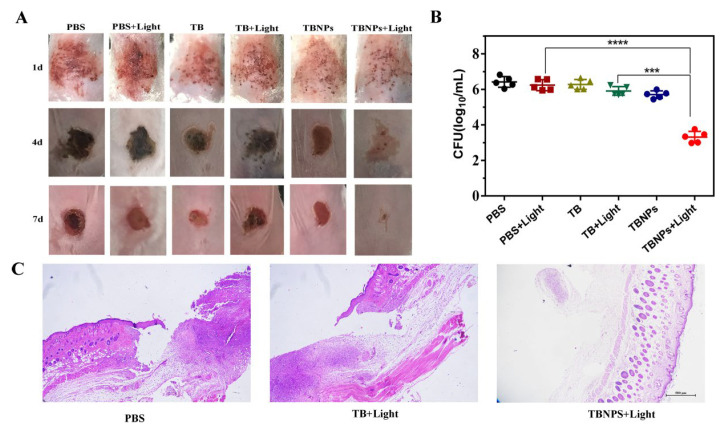
(**A**) Photographs of infected wounds in mice at different time points after various treatments. (**B**) Numbers of viable MRSAs in infected wounds after different treatments determined by the plate counting method. (**C**) Photomicrographs of tissue sections of MRSA-infected wounds in mice treated with PBS, TB + light, and TBNPs + light after H–E staining. The scale bar corresponds to 500 μm. **** very significant difference, *** significant difference.

An animal model of skin infection induced by MRSA was established to confirm the antibacterial effects of TBNPs under laser irradiation. The infected BALB/c mice were divided into six groups (PBS, PBS + light, TB, TB + light, TBNPs, and TBNPs + light; five mice per group). The BALB/c mice in the laser treatment group were irradiated with the 660 nm laser at 1 W/cm^2^ for 5 min. The appearance of the skin of each mouse was recorded every three days. In the gross examination, after the treatment for 1 day, swelling and red exudates were observed in the wound skin in the PBS, PBS + light, TB, and TB + light groups. In contrast, no obvious swelling or exudates were observed in the wounded skin in the TBNPs + light group. After treatment for 4 days, the wounds of the other groups were black or red, while the wounds of the TBNPs + light group were pale red. The areas of wounded skin were significantly smaller in the TBNPs + light group than in the others. On the seventh day after infection, the wound of the TBNPs + light group was well healed, with no significant crusting or ulceration, while the other groups formed scabs with different sizes (Figure 5A). In the histological examination, the TBNPs + light group did not exhibit obvious skin abnormalities, while the other groups of mice exhibited an incomplete epidermal morphology, severe edema tissue, and a large amount of leukocyte infiltration in the wound and surrounding tissue (Figure 5C and Figure S10). This suggests that the TBNPs + light group therapy had a significant promoting effect on wound healing. Similarly, the body weights of each group of mice were recorded. The body weights of the animals in each group increased steadily (Figure S11).

Further, in the process of wound healing, the size of the infected wound was measured every 3 days. As shown in Figure S12, the infected wound areas of PBS, PBS + light, TB, TB + light, TBNPs, and TBNPs + light were significantly different. On day 1, the size of the wound skin in these groups was not significantly different. On days 3 and 7, the size of skin wounds in the TBNPs group was significantly smaller than those in the other groups. PLGA promotes angiogenesis and accelerates the wound healing. Therefore, TB + light promotes wound healing during the sterilization process.

MRSA colony counting was performed at the wound sites 1, 4, and 7 days post treatment (Figure S13). The MRSA value in the PBS group was similar to that in the PBS + light group, while those in the TB and TB + light groups were decreased by only 0.14 log and 0.50 log, respectively (Figure 5B). The restraining effect of TBNPs on MRSA under laser irradiation can reach approximately 3 logs, which indicates that the photothermal enhancement therapy of TBNPs has a significant bactericidal effect on wounds infected by MRSA compared to that of the other groups. Consistent with the wound healing results, the bacteria in the TBNPs + light group were significantly reduced. Therefore, the laser irradiation of TBNPs can significantly inhibit the bacterial growth and promote wound healing.

### 3.7. Antibacterial Mechanism of TBNPs

**Figure 6:**
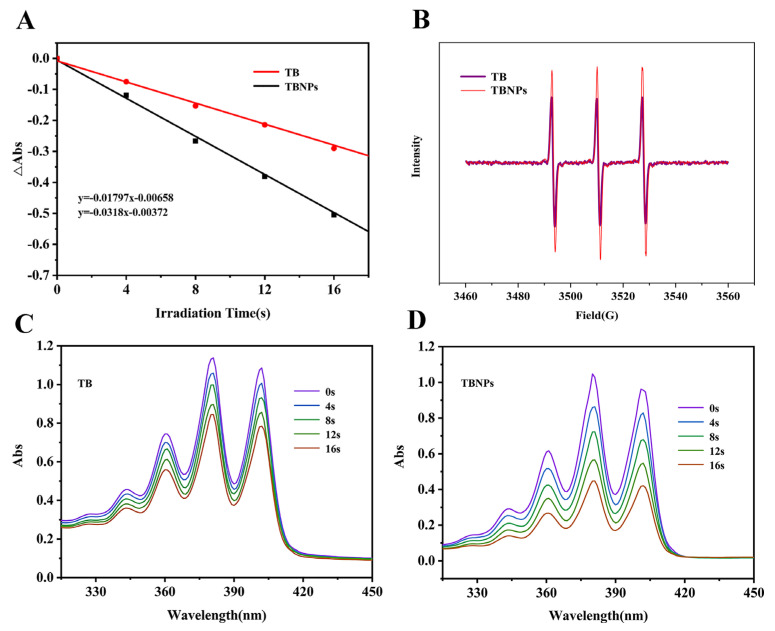
(**A**) Decay plots of ABDA absorption in the presence of TB and TBNPs in water. (**B**) EPR spectra of TB and TBNPs. Absorption spectra of ABDA mixed with (**C**) TB and (**D**) TBNPs in an aqueous solution with 660 nm laser irradiation.

In vitro and in vivo experiments confirmed that TBNPs have excellent antibacterial effects under laser irradiation. We suspected that TBNPs had a good antibacterial effect under laser irradiation because TBNPs produced more ^1^O_2_. TB was used as a reference agent, while ABDA was used as a ^1^O_2_ scavenger to test the ^1^O_2_ produced by TBNPs under laser irradiation. The significant decrease in ABDA absorption demonstrated the good ^1^O_2_ generation ability of TBNPs in water (Figure 6A). As shown in Figure 6B, the decomposition rate of ABDA in the presence of TBNPs is considerably higher than that of TB. EPR and ESR analyses also showed a better capacity for the generation of ^1^O_2_ by TBNPs compared to TB under 660 nm laser irradiation (Figure 6C,D).

In this study, the hypothetical antibacterial mechanism of TBNPs + light therapy is that ^1^O_2_ produced by TBNPs is more than that of TB under 660 nm irradiation, which is due to the reduced stack-up effect of ^1^O_2_ produced by TBNPs.

## 4. Discussion

Bacterial infections are the most common complication of wounds and account for one-third of global mortality [28]. However, the excessive use of antibiotics has led to the emergence of drug-resistant bacteria, posing a serious threat to public health [29]. Thus, the development of effective treatment modalities is of paramount significance.

PDT is considered a promising antibacterial method. Numerous studies have been carried out on PDT using visible light and different photosensitizers [30,31,32]. To obtain a better bactericidal effect, photosensitizers with a high affinity for bacterial cells have been investigated. TB, as a traditional photosensitizer, is limited in clinical applications due to the ACQ effect [33]. The antibacterial effect of PDT is generally attributed to the formation of ROS that do not induce drug resistance [34]. The purpose of this study was to investigate the antibacterial effect of TB and TBNPs and provide an effective method to solve global health problems.

In this study, TBNPs were successfully prepared using an ultrasound-assisted coating method and characterized. In the ultraviolet–visible spectrum, the TBNPs exhibited a characteristic peak at 660 nm, which is consistent with previous reports [35,36]. According to the FTIR results, TB was successfully encapsulated into the PLGA NPs. The encapsulation rate of TBNPs was 87.03%, which was higher than those of other nanomaterials reported in the literature [37]. The antibacterial experiment of TBNPs confirmed that TBNPs have excellent antibacterial effects, which may be related to the high TB encapsulation rate and PDT. ROS generation is the key to PDT. More ^1^O_2_ was detected in TB-irradiated cells than in unirradiated cells. The main target of ROS is the cell membrane, which can lead to protein leakage and lipid peroxidation, causing bacterial DNA breakage and, ultimately, bacterial death [38,39]. The ACQ effect is one of the factors affecting the application of nanomaterials [40]. In recent years, many hydrophobic counterions have been proposed as effective structures to reduce the ACQ. However, the role of the counterion structure is still poorly understood [41]. As a hydrophobic counterion material, PLGA increases the encapsulation efficiency of dyes [42]. Numerous PDT nanomaterials are used in antibacterial applications owing to their unique antibacterial effects. Porphyrin-mediated PDT may kill bacteria by destroying the cell wall and membrane of bacteria [43], while ICG-PDT can thin the cell wall of MRSA and drive the transformation of MRSA to the sensitive phenotype of oxacillin, which can achieve excellent results in coordination with antibiotic use [44]. In addition, PDT nanomaterials have a significant role not only in the antibacterial activity, but also in the antifungal and antiparasitic activities. Phenothiazines, phthalocyanines, porphyrin, and porphyrinic precursor aminolevulinic acid plus PDT fungi have a positive role in the treatment [45]. PLGA prepared in this study coated the TB, increased the encapsulation rate of TB, largely reduced the ACQ effect, and improved the antibacterial effect of TBNPs. In addition, PLGA can promote the function of TBNPs in the anti-infection repair of injured skin because of its ability to promote angiogenesis and wound healing.

This study confirmed that PLGA-coated TBNPs had significant antibacterial effects against MRSA and *E. coli*. TBNPs have higher binding efficiency and ROS production than free TB, which suggests that it enhances the PDT. The enhanced activity is attributed to the relatively high loading capacity and the TBNPs attenuating the ACQ effect of TB, which reveals the advantage of TBNPs in PDT.

## 5. Conclusions

In this study, a PLGA-coated TB antibacterial nanomaterial was successfully synthesized, which has a photodynamic effect and produces more ROS, with a high antibacterial ability. TBNPs are broad-spectrum antibacterial materials with a good antibacterial activity against MRSA and *E. coli* with synergistic PDT. TBNPs can reduce the ACQ effect of TB, thus increasing the PDT effect of TB. In addition, TBNPs can produce more ROS in coordination with PDT to achieve better antibacterial effects. In vivo animal studies further demonstrated that the TBNPs could promote wound healing by reducing inflammation and increasing the collagen deposition and blood vessel formation. The TBNPs obtained in this study are biocompatible and hemocompatible, having a broad spectrum of antibacterial activity, and can be used as a safe and curable alternative for the treatment of chronic, persistent skin injuries and drug-resistant bacterial infections.

## Data Availability

The data presented in this study are available on request from the corresponding author.

## Supplementary Materials

The following are available online at https://www.mdpi.com/article/10.3390/nano11123394/s1, Figure S1 Hydration diameter distribution of TBNPs in water. Figure S2 The DLS analysis of TBNPs in water. Figure S3 Zeta of TBNPs in pure water. Figure S4 TBNPs SEM images. Figure S5 Restrain rate of different groups against *E. coli* and MRSA. Figure S6 Hemolysis of red blood cells (RBCs) treated with control, TBNPs (10, 50, 100, and 400 µg mL^−1^) and Triton-X100 solution. Figure S7 Hemolysis percentage of red blood cells (RBCs) treated with TBNPs solution. Figure S8 The photograph under a microscope of red blood cells (RBCs). Figure S9 Weight curve of mice within 7 days after mice was injected different groups (PBS, PBS + light, TB, TB + light, TBNPs and TBNPs + light). Figure S10 Activities of 4T1 cells incubated with TB and TBNPs at different concentrations. Figure S11 Photomicrographs of tissue sections of MRSA-infected wounds in mice treated with TB and TBNPs after H&E staining. The scale bar is 500 µm. Figure S12 The relative wound area in different groups (PBS group, PBS + light group, TB group, TB + light group, TBNPs group and TBNPs + light group). Figure S13 Photographs of bacterial colonies formation on LB agar plates from the infected wounds after 7 d treatments.
