# Supplementary material for: Poly Lactic-co-Glycolic Acid-Coated Toluidine Blue Nanoparticles for the Antibacterial Therapy of Wounds
Source: Nanomaterials (Basel). 2021 Dec 14;11(12):3394. doi: 10.3390/nano11123394 (PMC8708285; doi:10.3390/nano11123394)
Supplement: Supplementary file 1 [file nanomaterials-11-03394-s001.zip › nanomaterials-1450954-supplementary.pdf]

nanomaterials

Supplementary Materials

Poly Lactic-*co*-Glycolic Acid-Coated Toluidine Blue Nanoparticles for the Antibacterial Therapy of Wounds

Xiaomu Xu †, Bo Liu †, Haiyan Wu, Yichi Zhang, Xinyuan Tian, Jijing Tian * and Tianlong Liu *

Laboratory of Veterinary Pathology and Nanopathology, College of Veterinary Medicine, China Agricultural University, , No. 2 West Road Yuanmingyuan,
Beijing 100193, China; xuxiaomu92@126.com (X.X.); liubo19900409@163.com (B.L.); wuhaiyan_helen@sina.com (H.W.); zhangyichi142857@163.com (Y.Z.); tianxy@cau.edu.cn (X.T.)
* Correspondence: jjtian@cau.edu.cn (J.T.); liutianlong@cau.edu.cn (T.L.); Tel.: +86-010-62733398 (T.L.)
† These authors contributed equally to this work.

The formula of entrapment efficiency (EE) and drug loading (DL)

M1 is the mass of TB added in the preparation of nanoparticles, C is the concentration of TB in the supernatant, V is the volume of supernatant, M2 is the mass of PLGA added in the preparation of nanoparticles.

$$EE= (M1- C\cdot V) / M1$$

$$DL= (M1- C\cdot V) / [(M1- C\cdot V) + M2]$$

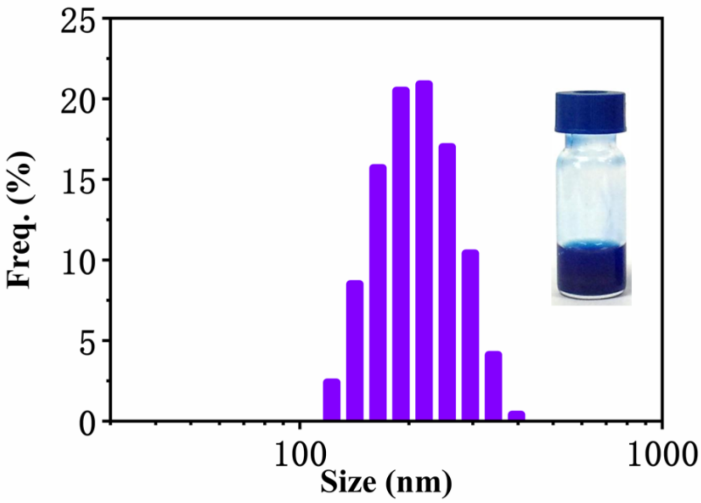

Figure S1. Hydration diameter distribution of TBNPs in water.

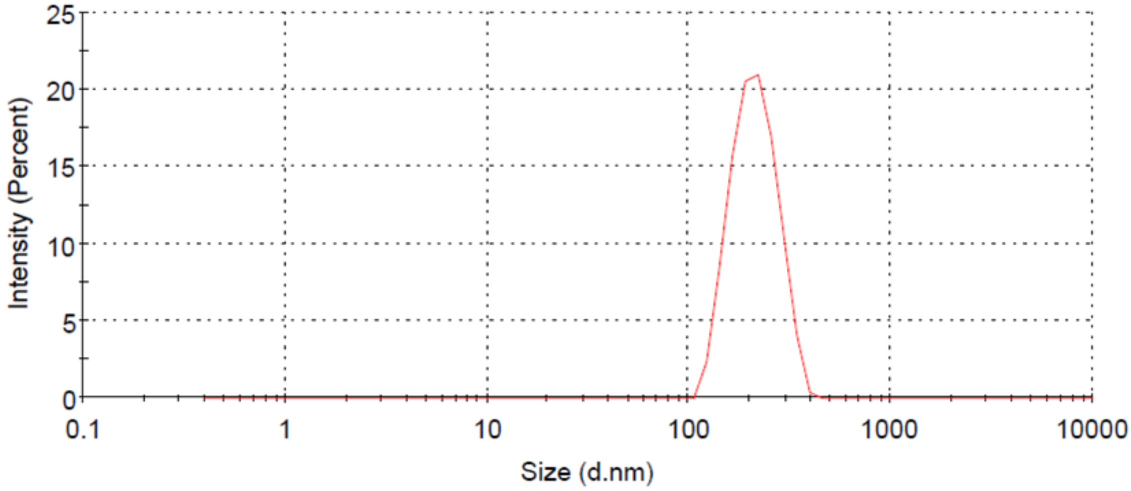

Figure S2. The DLS analysis of TBNPs in water.

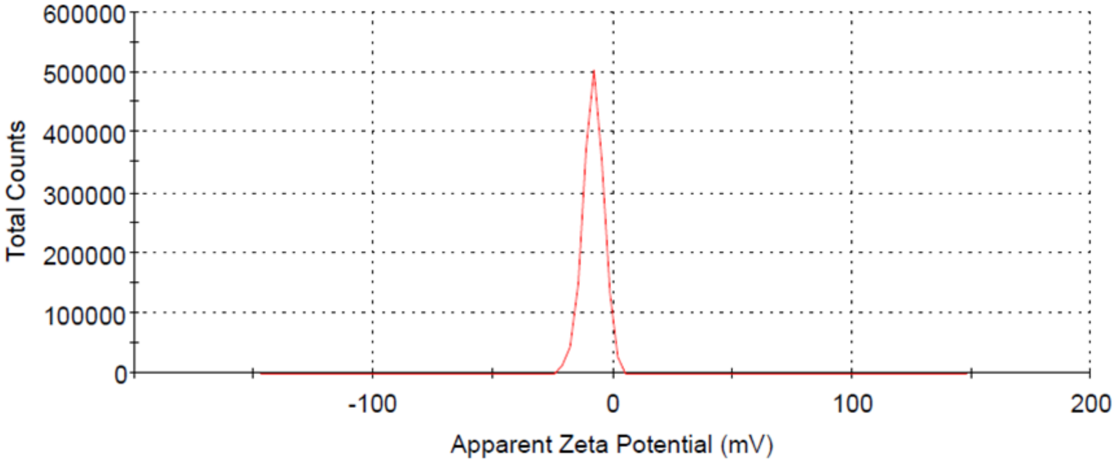

Figure S3. Zeta of TBNPs in pure water.

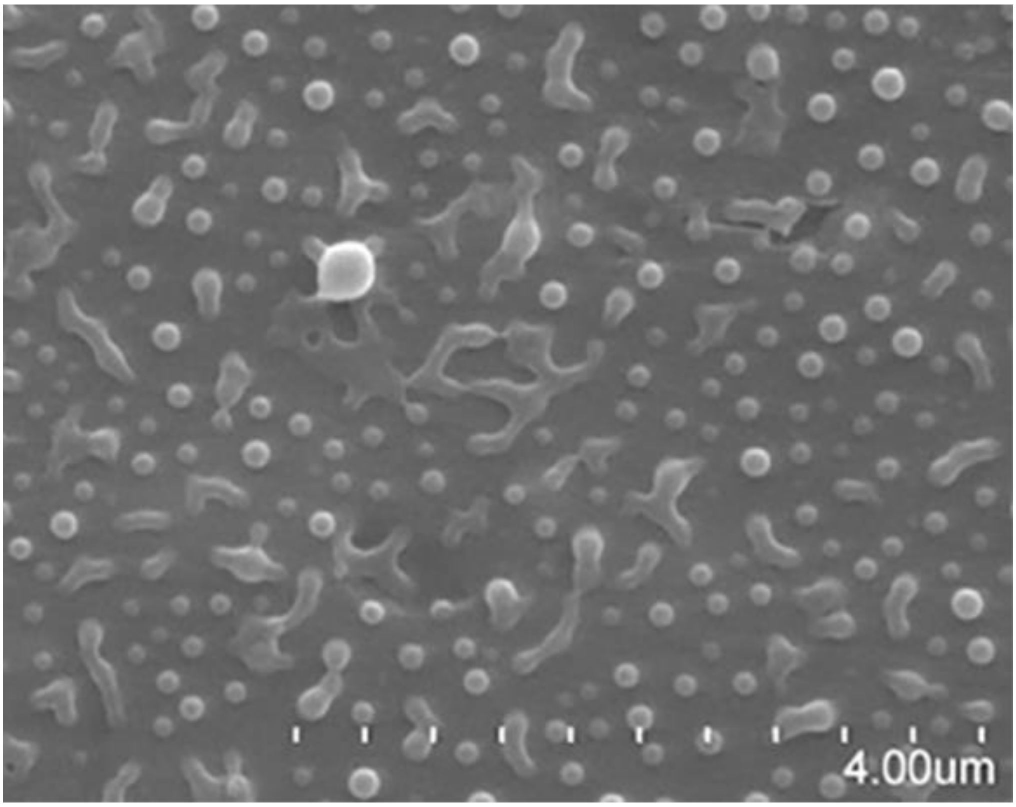

Figure S4. TBNPs SEM images.

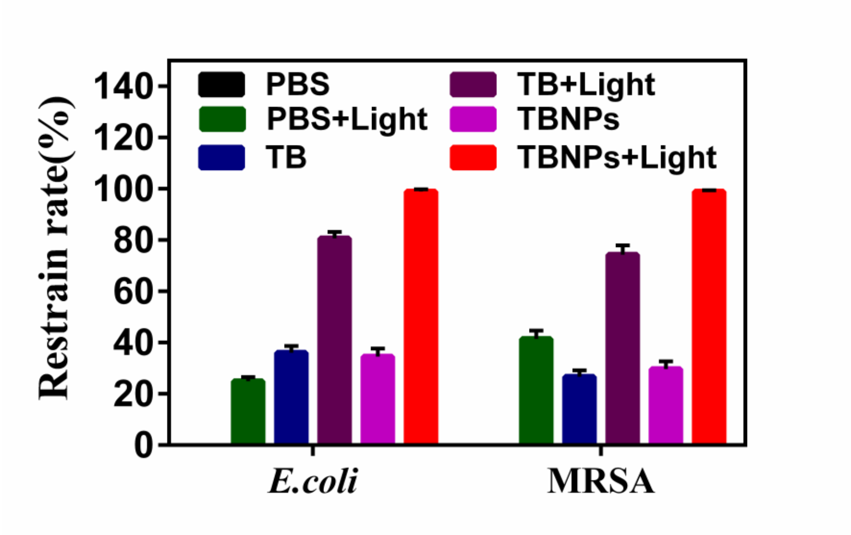

Figure S5. Restrain rate of different groups against *E. coli* and MRSA.

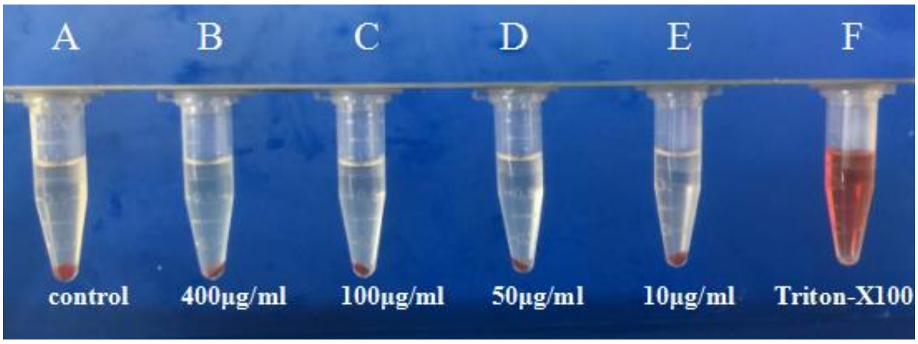

Figure S6. Hemolysis of red blood cells (RBCs) treated with control, TBNPs (10, 50, 100, and 400 μg mL^{-1}) and Triton-X100 solution.

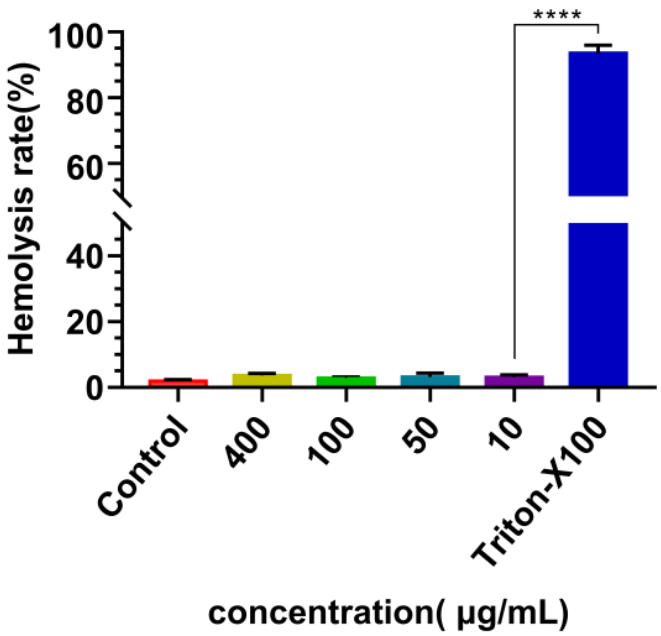

Figure S7. Hemolysis percentage of red blood cells (RBCs) treated with TBNPs solution.

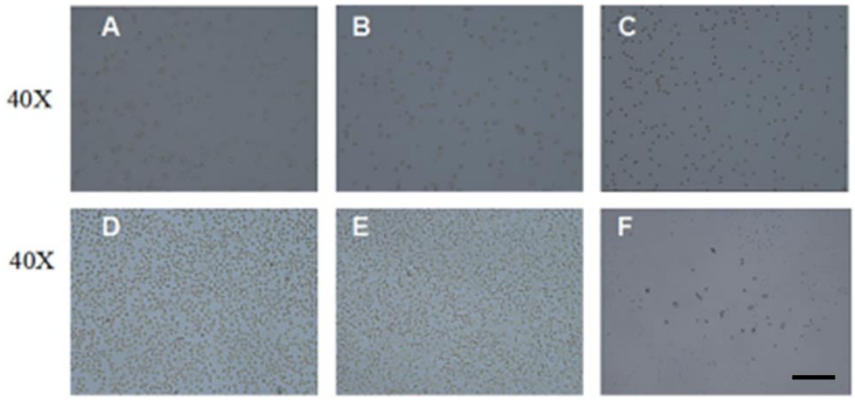

Figure S8. The photograph under a microscope of red blood cells (RBCs) .

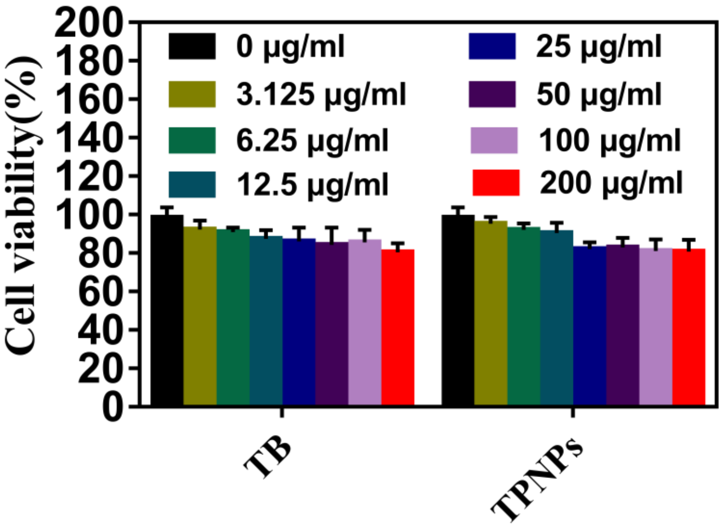

Figure S9. Activities of 4T1 cells incubated with TB and TBNPs at different concentrations.

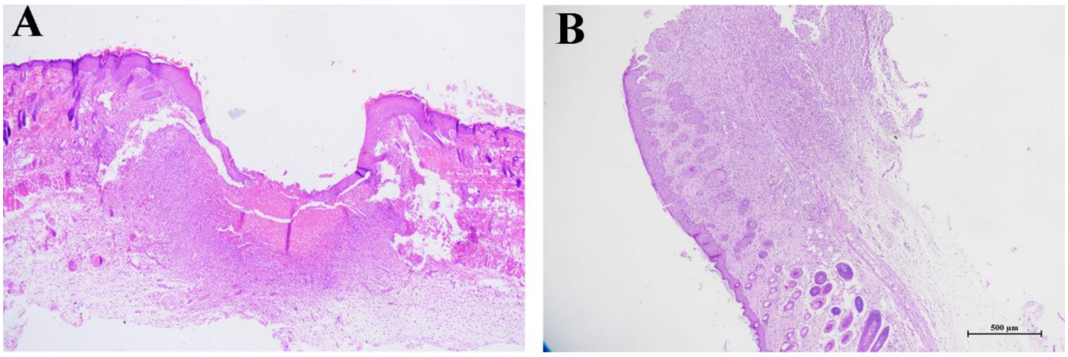

TB TBNPS

Figure S10. Photomicrographs of tissue sections of MRSA infected wounds in mice treated withTB, and TBNPs after H&E staining. The scale bar is 500 μm.

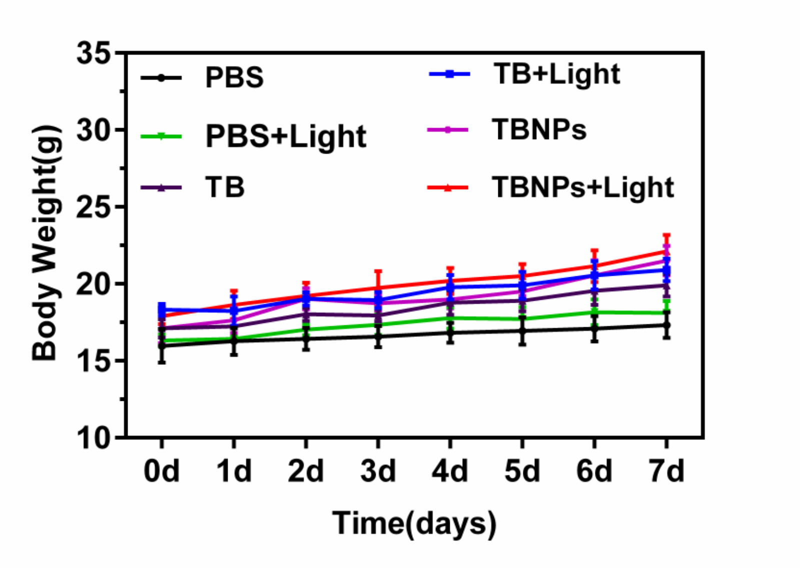

Figure S11. Weight curve of mice within 7 days after mice was injected different groups.(PBS, PBS+Light, TB, TB+Light, TBNPs and TBNPs+Light).

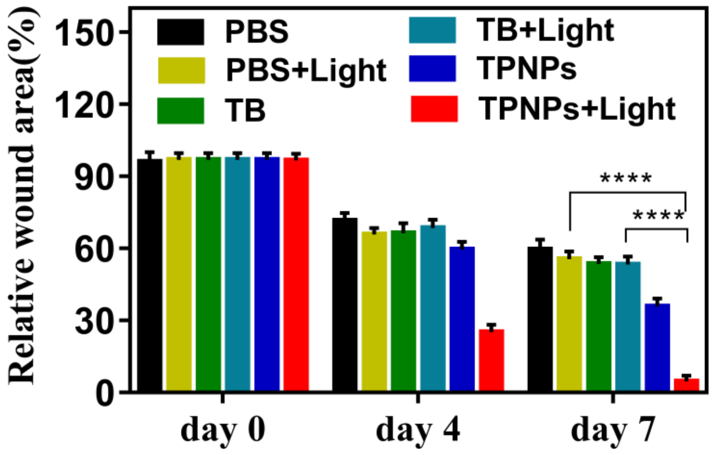

Figure S12. The relative wound area in different groups (PBS group, PBS+Light group, TB group, TB+Light group, TBNPs group and TBNPs+Light group).

PBS PBS+Light TB TB+Light TBNPs TBNPs+Light

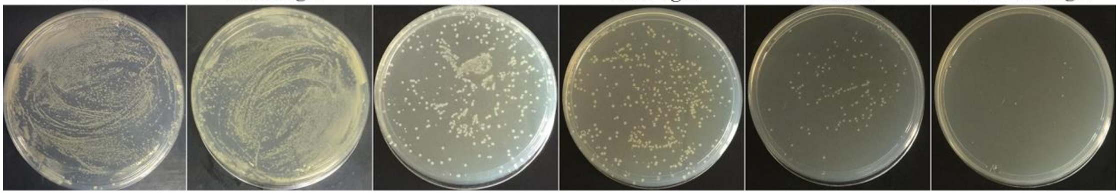

Figure S13. Photographs of bacterial colonies formation on LB agar plates from the infected wounds after 7d treatments.
